# Digital subtraction imaging with carbon dioxide and liquid contrast in the biliary and pancreatic ducts

**DOI:** 10.1055/a-2589-1094

**Published:** 2025-05-09

**Authors:** Akihiro Maruyama, Makoto Kobayashi, Hirotaka Takeshima, Hiroshi Nakayabu, Hiroki Kato, Shintaro Tominaga, Motoyoshi Yano

**Affiliations:** 1 Department of Gastroenterology, Yokkaichi Municipal Hospital, Yokkaichi, Mie, Japan

Digital subtraction imaging (DSI) is used to enhance visualization 1 2 3 . By subtracting a precontrast image from a postcontrast image, DSI identifies anatomical structures while minimizing interference from surrounding tissues. It enhances the evaluation of biliary and pancreatic ducts by removing background structures and improving image clarity. This report presents two cases demonstrating the utility of CO _2_ digital subtraction cholangiography (CDDSC) and contrast digital subtraction pancreatography (CDSP) in clinical practice. An Ultimax-i DREX-U180 (Canon) was used to acquire the DSI sequences. Case 1 involves a 66-year-old woman with hilar cholangiocarcinoma. To evaluate biliary anatomy, CDDSC was performed via an endoscopic nasobiliary drainage tube with CO _2_ infusion at a controlled rate of 1 mL/s. CO _2_ allows clear visualization of separate intrahepatic ducts, which is challenging with traditional liquid contrast agents ( Fig. 1 ). Rapid absorption of CO _2_ by the body reduces the risk of postprocedural complications such as cholangitis. This technique successfully delineated tumor-induced strictures, enabling precise assessment of biliary obstruction and facilitating effective treatment planning. Case 2 is a 71-year-old woman evaluated for pancreatic duct stenosis. CDSP was performed using an endoscopic nasopancreatic drainage tube, with amidotrizoic acid infused at 0.4 mL/s. Vertebral interference was effectively removed, and visualization of the pancreatic body and branch ducts was enhanced ( Fig. 2 ). Improved image clarity enabled a detailed assessment of the stenotic segment, potentially allowing differentiation between benign and malignant changes. The total radiation dose was 9.4 mGy in Case 1 and 8.5 mGy in Case 2. Both cases highlight the advantages of DSI ( Video 1 ). CDDSC is a safer alternative to biliary imaging, reducing procedural complications while providing high-resolution images. Similarly, CDSP improves pancreatic duct evaluation by overcoming anatomical challenges and enhancing diagnostic accuracy. These imaging techniques are safer, more effective, and a substantial progress in the evaluation of ductal systems.

**Fig. 1:**
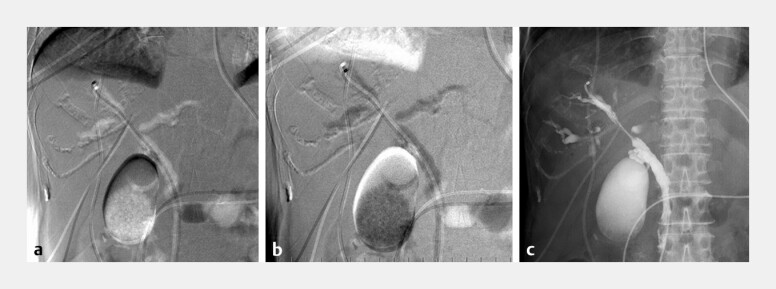
**a** Radiograph obtained 3 seconds after the infusion of CO _2_ at 1 mL/s via an ENBD tube during digital subtraction imaging. **b** A radiograph showing the same image as (a), but with inverted black-and-white contrast. **c** Radiograph obtained 6 seconds after the infusion of amidotrizoic acid at 1 mL/s via an ENBD tube. Abbreviation: ENBD, endoscopic nasobiliary drainage.

**Fig. 2:**
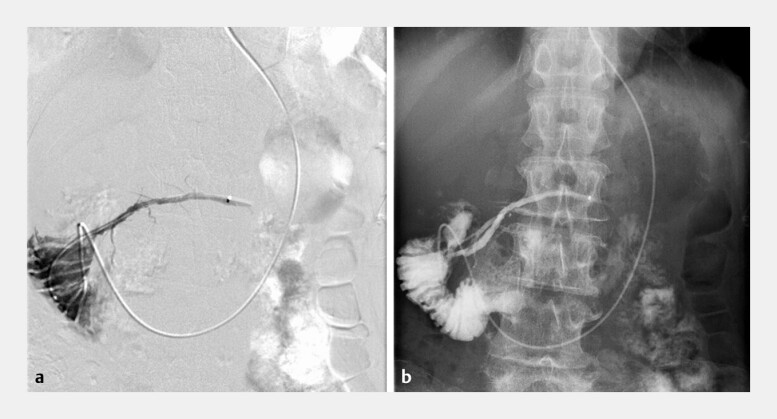
**a** Radiograph obtained 3 seconds after the infusion of amidotrizoic acid at 0.4 mL/s during digital subtraction pancreatography. **b** Radiograph obtained 3 seconds after the infusion of amidotrizoic acid at 0.4 mL/s without digital subtraction imaging.


This video demonstrates the application of digital subtraction imaging (DSI) for enhanced visualization of biliary and pancreatic ducts. CO _2_ was infused at 1 mL/s via an endoscopic nasobiliary drainage (ENBD) tube, effectively contrasting the hilar bile ducts due to its diffusibility and rapid absorption. Subsequently, an iodinated contrast agent was infused at the same rate, which provided less immediate visualization due to higher viscosity. For pancreatic imaging, the iodinated contrast agent was infused at 0.4 mL/s via an endoscopic nasopancreatic drainage (ENPD) tube. DSI subtracted overlapping vertebral structures, enabling clearer visualization of branch pancreatic ducts in the pancreatic body. These results highlight the potential of DSI in achieving precise and detailed imaging for biliary and pancreatic evaluations.
Video 1

Endoscopy_UCTN_Code_TTT_1AR_2AB

## Notes

Endoscopy E-Videos https://eref.thieme.de/e-videos
*E-Videos* is an open access online section of the journal *Endoscopy* , reporting on interesting cases and new techniques in gastroenterological endoscopy. All papers include a high-quality video and are published with a Creative Commons CC-BY license. Endoscopy E-Videos qualify for HINARI discounts and waivers and eligibility is automatically checked during the submission process. We grant 100% waivers to articles whose corresponding authors are based in Group A countries and 50% waivers to those who are based in Group B countries as classified by Research4Life (see: https://www.research4life.org/access/eligibility/ ). This section has its own submission website at https://mc.manuscriptcentral.com/e-videos .
